# A Rare Case of Squamous Cell Carcinoma of the Bladder Presenting as a Metastatic Right Ventricular Mass

**DOI:** 10.1155/2010/789609

**Published:** 2010-03-17

**Authors:** Joanna M. Bonsall, Rhome Hughes, Mario Mosunjac, David Harrison, Habib Samady

**Affiliations:** ^1^Department of Medicine, Emory University School of Medicine, Atlanta, GA 30332, USA; ^2^Department of Pathology, Emory University School of Medicine, Atlanta, GA 30332, USA

## Abstract

A 74-year-old woman presented with bilateral lower extremity swelling, worsening dyspnea on exertion, and mild hemoptysis. An echocardiogram at time of admission showed a mass in the right ventricle. The pathology of a sample obtained via transvenous biopsy was consistent with squamous cell carcinoma; no primary source could initially be identified. Severe thrombocytopenia, likely consumptive, precluded surgical intervention, so the patient underwent palliative radiation. Unfortunately, she developed fatal respiratory failure. Upon autopsy, the bladder was found to contain polyps of invasive squamous cell carcinoma, similar in morphology to the tumor mass in the heart. Her lungs contained multiple tumor emboli at different stages, which was likely the final cause of her death. Squamous cell carcinoma metastases to the endocardium are extremely rare and without defined treatment. Surgery can improve prognosis in those with primary tumors that are benign or without metastases. In those with symptomatic metastatic tumors, palliative debulking can done although generally will not improve prognosis. It is currently unknown whether radiation improves survival. In this case, irradiation did destroy a portion of the tumor as the final pathology showed extensive necrosis of the tumor; unfortunately, it did not change her symptoms and did not change the final outcome.

## 1. Introduction

Squamous cell carcinomas metastases to the endocardium are rare and, when occurring as a right ventricular mass, can present with right ventricular outflow tract obstructive symptoms and can also shed emboli into the pulmonary circulation [1]. Here we discuss a case of a patient who presented with right heart failure and pulmonary emboli, whose ultimate diagnosis was metastatic squamous cell carcinoma of the bladder. In the past twenty years, there have been only two reports of squamous cell carcinoma in the heart that originated from the bladder [2, 3]; this is the first case of the cardiac mass being the presenting symptom.

## 2. Presentation

A 74-year-old woman presented with a 6-month history of bilateral lower extremity swelling and worsening dyspnea on exertion. She also reported some episodes of mild cough with scant hemoptysis over the same time course. She had no chest pain, orthopnea, or paroxysmal noctural dyspnea. She had no weight loss or fevers. She had no known exposure to any infectious diseases and had not traveled outside the United States. Her past medical history was only significant for noninsulin dependant diabetes and hypertension, both diagnosed within the past year, and a remote history of kidney stones. Her medications were Hydrochlorothiazide, Metformin, and Aspirin. She had never used tobacco and drank alcohol only occasionally. 

She had presented to an outside hospital with these complaints approximately two weeks prior to admission to our facility. On her initial evaluation at the outside hospital, she underwent an echocardiogram and a cardiac catheterization, which were normal. She was discharged home but returned to the outside hospital within two weeks. On the second admission, she had a CT of her chest with contrast which showed a filling defect in the right ventricle that extended into the right pulmonary outflow tract, occlusions of bilateral segmental pulmonary arteries, a small to moderate pericardial effusion, and small bilateral pleural effusions. A follow-up echocardiogram confirmed a right ventricular mass. She was transferred to our facility for further workup and treatment. 

On physical exam, the patient was afebrile, her blood pressure was 103/56, she had a pulse rate of 86 and a respiratory rate of 16, and her oxygen saturation was 96% on 2 liters of oxygen via nasal cannula. She was observed to be dyspneic on minimal exertion. Her jugular venous pulse was elevated to 12 cm of water. She had no heaves. She had a normal S1 and S2 with no S3 or S4 but did have a 3/6 holosystolic murmur at the left lower sternal border radiating to the right sternal border, which increased with inspiration. She had 1+ pitting edema in her lower extremities and good distal pulses. Her pulmonary, abdominal, and neurologic exams were normal. She had no petechiae or gingival bleeding. She had a Foley catheter in place which had been inserted upon admission; the urine was dark yellow with no hematuria noted. Significant labs upon admission were a sodium level of 132 mEq/L and an albumin of 3.0 mg/dL. Her creatinine was normal. Her hemoglobin was 10.9 gm/dL and hematocrit was 32%, with an MCV of 83.5 fL. Her platelet count was 54,000 cells/mcL; her platelet count the following morning dropped to 16,000 cells/mcL. She had a white cell count of 14,600 cells/mcL with 77% granulocytes. Her urinalysis, drawn from the Foley catheter, was found to have large numbers of squamous cells and a urobilinogen of 8 EU. The remainder of her urinalysis was normal. Her heparin-induced-thrombocytopenia (HIT) antibody panel was negative. Her EKG was notable for a right bundle branch block. 

**Figure 1:**
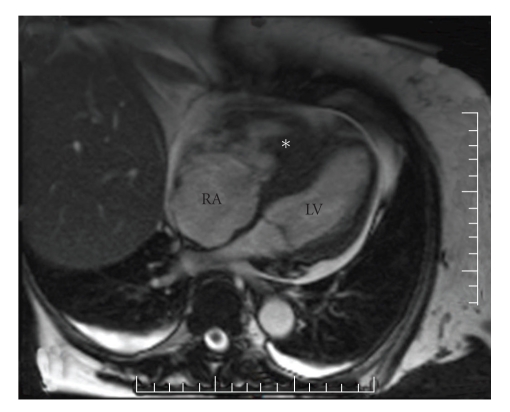
A magnetic resonance image (with gadolinium) shows a transverse view of the heart in diastole. The mass in the right ventricle (*asterisk*) fills most of the ventricle, and impedes filling of the left ventricle (LV). Note also the severe dilatation of the right atrium (RA) as well as the pericardial effusion (bright).

An echocardiogram performed on admission confirmed that the right ventricular mass, which extended into the right ventricular outflow tract, was attached to the septum, and had mobile components. Cardiothoracic surgery was consulted urgently for possible surgical assessment and/or removal of the mass; however, her thrombocytopenia made her perioperative mortality risk unacceptably high. An MRI showed a right ventricular avascular mass originating from the intraventricular septum, along with multiple nodular irregularities seen on the visceral pericardium and a moderate pericardial effusion (Figure 1). Other imaging studies included lower extremity Doppler ultrasounds showed no thromboses, and a CT of the abdomen and pelvis, did not show any masses suspicious of neoplasm or any lymphadenopathy. 

The working diagnosis at this point was that the mass was a malignancy with potentially overlying thrombus. The decision was made to treat the patient with alteplase (recombinant tissue-type plasminogen activator, tPA) in the hopes of dissolving any potential clot, to be followed with a biopsy of any mass that remained. The patient was treated with tPA introduced through a catheter threaded into the right ventricle. A follow-up echocardiogram did not show any appreciable reduction in the size of the mass. A transvenous biopsy of the mass was subsequently performed under ultrasound guidance. 

## 3. Diagnosis

The differential diagnosis of a ventricular mass includes tumors as well as “pseudotumors” such as thrombi or lipomas [4]. Tumors in the ventricle are most commonly benign and include myxomas, papillary elastomas, and lipomas [5]. When malignant, tumors are most likely to be metastatic in origin, spreading to the heart through direct extension via lymphatics or blood stream [1]. The most common metastatic tumors in the heart originate from common malignancies such as carcinoma of the lungs, breast, stomach, liver, and colon, as well as leukemias/lymphomas [1, 6]. Some rarer cancers, such as melanoma and renal cell carcinoma, also have a relatively high incidence due to their predilection for the heart; 30%–50% of melanoma patients will have cardiac metastases discovered at autopsy [6]. Overall, the reported incidence of cardiac metastases in those with known prior malignancies ranges from 2.3%–18.3%; many cases are asymptomatic and found at autopsy [1]. 

A typical approach to a cardiac mass in the endocardium involves imaging prior to surgical sampling. Transthoracic echocardiography can provide details of the mass such as location, shape, and mobility. Cardiac MR can be used to further characterize the mass and can distinguish between fat, fluid, or blood products, which can be helpful in distinguishing between various primary tumors. Tissue plane delineation is also better in CMR than TTE and can determine the exact attachment sites as well as any potential invasion [7]. For right-sided tumors, such as in this patient, a TEE-guided transvenous biopsy is recommended after imaging [6]. 

In our patient, the pathological diagnosis of the right ventricular mass was consistent with a metastatic squamous cell carcinoma of unknown primary site. A follow-up PET scan with 18F-FDG showed only the lesion in the right ventricle, a mildly hypermetabolic lymph node which was judged to be likely reactive, and a small hypermetabolic area of skin thickening over the right posterior shoulder which could not be clinically correlated with any lesion. 

## 4. Management

Squamous cell carcinoma metastases to the endocardium are extremely rare and the treatment has not been clearly defined. In other cases of right ventricular tumors, surgery can improve prognosis in those with benign tumors or with primary malignant disease with no evidence of metastases [6]. In those with metastatic tumors that are symptomatic, palliative debulking can ease the symptoms, although it will not generally improve prognosis. Surgery should be performed urgently when the potential exists for catastrophic embolization [6]. Surgery is not recommended for those with wide-spread disease whose symptoms are minor. Our patient was not a surgical candidate due to her thrombocytopenia, so we began radiation in the hopes of symptomatic relief, with no expectation of cure. Unfortunately, her heart failure and thrombocytopenia continued to worsen, and she also developed renal failure. Twenty-five days after she was admitted to the hospital, she developed acute respiratory failure and, in accordance with her family's wishes, it was not resuscitated. The family agreed to an autopsy.

**Figure 2:**
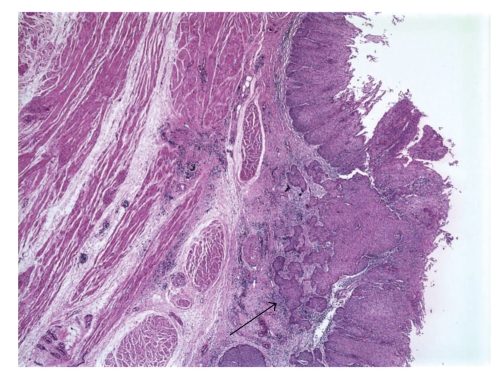
At autopsy, the bladder mucosa was found to have diffuse squamous metaplasia. A microscopic image demonstrates a region of invasive squamous cell carcinoma (*arrow*) that extended into the muscularis propria.

Squamous cell carcinoma metastases to the endocardium/intraventricular cavity are rare. In the past 5 years, there have been 10 case reports of squamous cell carcinoma metastases in the right ventricle [2, 8–17]. Of these, 5 originated from the cervix [9–13]; others originated from the tongue [8], larynx [14], maxillary sinus [16], esophagus [17], and bladder [2]. Presentations typically included weight loss, heart failure, fatigue and dyspnea, and syncope. Several patients had pulmonary emboli at the time of presentation. Treatments ranged from surgical resection to radiation to chemotherapy. All of the cases had a known history of malignancy. Of the outcomes reported, all except one (in whom no follow-up was described) died within days to at most 5 months after presentation, regardless of therapy. In our patient, although no evidence of a primary lesion was found on PET scan or CT scan, at the time of autopsy, three 0.5 to 1.0 cm mucosal based papillary lesions were identified at the bladder base. On microscopic examination, these lesions were identified as moderately-differentiated squamous cell carcinoma, invasive to the level of the muscularis propria, arising in a background of extensive squamous metaplasia and dysplasia of the bladder urothelium (Figure 2). 

**Figure 3:**
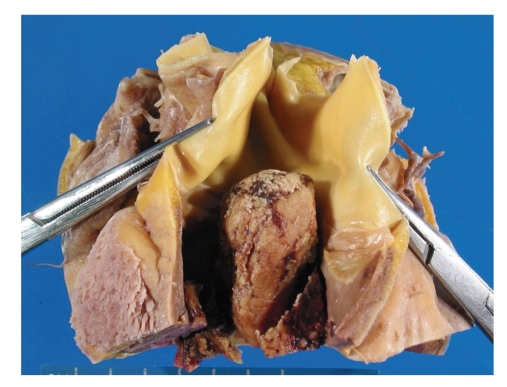
A postmortem view of the right ventricle shows the cardiac mass extending into the right ventricular outflow tract (held open by forceps).

The cardiac tumor mass identified within the right ventricle at the time of autopsy was 7 cm in the greatest dimension and had diffusely infiltrated into the septal wall and extended past the pulmonic valves (Figure 3). 

**Figure 4:**
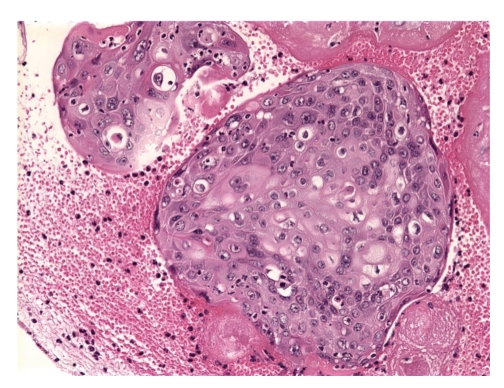
The tumor mass within the right ventricle exhibited features of squamoid differentiation, with extensive associated necrosis secondary to radiation. Additional nests of intravascular squamoid tumor cells within the myocardial vasculature were also observed.

Microscopic analysis showed well to moderately differentiated squamous cell carcinoma with extensive tumor necrosis. The morphology of the tumor cells was identical to the morphology those found in the bladder mucosa (Figure 4).

Her lungs contained multiple tumor emboli admixed with fibrin deposition at different stages of organization that were firmly adherent to the endothelial layer of the vessels. The largest pulmonary fresh embolus measured was 2.0 cm, and was likely the final cause of her demise. 

Of the ten cases of squamous cell cancer with cardiac metastases discussed above, only one originated from the bladder [2]; in fact, in the past twenty years, there were only two case reports of squamous cell carcinoma in the heart that originated from the bladder [2, 3]. Both of the cases had known diagnoses of bladder cancer prior to presentation; ours is the first case report of heart failure presenting as the initial symptom. Both patients died shortly after presentation; one died of pulmonary compromise from the cardiac mass [2] and the other died of a massive pulmonary embolus and was only discovered to have cardiac metastases at autopsy [3].

## 5. Conclusion

This report is the only reported case of a cardiac mass being the presenting symptom of metastatic squamous cell carcinoma of the bladder. This case also illustrates the difficulty of treating metastatic cardiac disease; surgery seems to be the preferred option but does not necessarily improve overall prognosis, although it may ease the symptoms. This patient presented with symptoms of right-sided heart failure from right ventricular outflow track obstruction, multiple bilateral tumor pulmonary emboli, and thrombocytopenia that was likely consumptive and unfortunately limited our treatment options. In this case, irradiation did seem to destroy a portion of the tumor as the final pathology showed extensive necrosis of the tumor; however, it did not improve her symptoms and did not change her final outcome.
